# Mimicking biological synapses with a-HfSiO_x_-based memristor: implications for artificial intelligence and memory applications

**DOI:** 10.1186/s40580-023-00380-8

**Published:** 2023-07-10

**Authors:** Muhammad Ismail, Maria Rasheed, Chandreswar Mahata, Myounggon Kang, Sungjun Kim

**Affiliations:** 1 Division of Electronics and Electrical Engineering, Dongguk University, Seoul, 04620 Republic of Korea; 2 Department of Electronics Engineering, Korea National University of Transportation, Chungju- si, 27469 Republic of Korea

**Keywords:** a-HfSiO_x_ film, Analog tunable switching, Excitatory postsynaptic current, Spiking-rate-dependent plasticity, Schottky emission

## Abstract

Memristors, owing to their uncomplicated structure and resemblance to biological synapses, are predicted to see increased usage in the domain of artificial intelligence. Additionally, to augment the capacity for multilayer data storage in high-density memory applications, meticulous regulation of quantized conduction with an extremely low transition energy is required. In this work, an a-HfSiO_x_-based memristor was grown through atomic layer deposition (ALD) and investigated for its electrical and biological properties for use in multilevel switching memory and neuromorphic computing systems. The crystal structure and chemical distribution of the HfSiOx/TaN layers were analyzed using X-ray diffraction (XRD) and X-ray photoelectron spectroscopy (XPS), respectively. The Pt/a-HfSiO_x_/TaN memristor was confirmed by transmission electron microscopy (TEM) and showed analog bipolar switching behavior with high endurance stability (1000 cycles), long data retention performance (10^4^ s), and uniform voltage distribution. Its multilevel capability was demonstrated by restricting current compliance (CC) and stopping the reset voltage. The memristor exhibited synaptic properties, such as short-term plasticity, excitatory postsynaptic current (EPSC), spiking-rate-dependent plasticity (SRDP), post-tetanic potentiation (PTP), and paired-pulse facilitation (PPF). Furthermore, it demonstrated 94.6% pattern accuracy in neural network simulations. Thus, a-HfSiO_x_-based memristors have great potential for use in multilevel memory and neuromorphic computing systems.

**Graphical Abstract:**

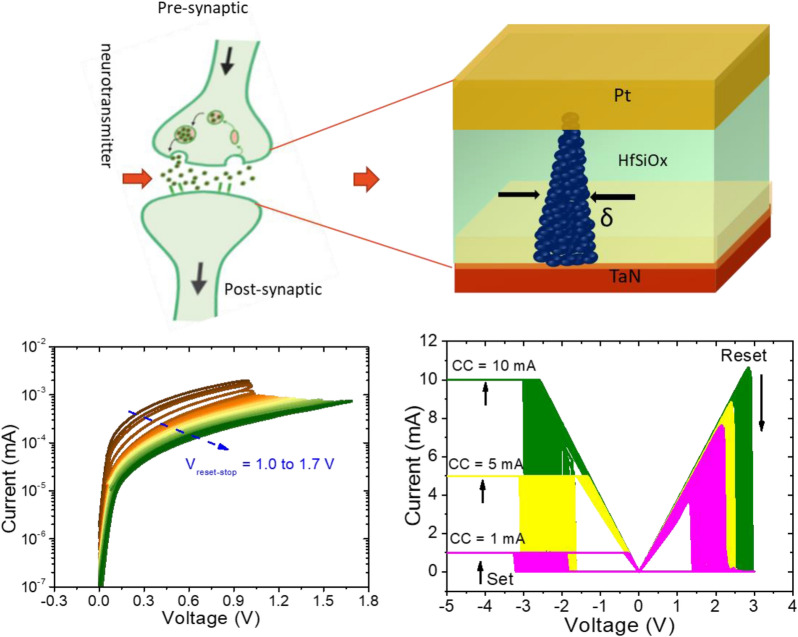

**Supplementary Information:**

The online version contains supplementary material available at 10.1186/s40580-023-00380-8.

## Introduction

Computers now must acquire, encrypt, and modify data and information with increasing efficiency due to the data and information explosion [1–3]. The von Neumann bottleneck refers to the performance limitation that arises in traditional computing architectures due to the separation of processing and memory. In this architecture, the CPU has to continuously retrieve data from memory to perform computations, which leads to a significant delay and limits the overall speed of the system. Memristors have the potential to address this issue as they can perform both memory and processing tasks within a single device, thus reducing the need for data movement between separate memory and processing units. This has led to the development of novel computing architectures, such as neuromorphic computing, that leverage the unique properties of memristors to achieve high computational efficiency and improved performance [4–7]. Although modern digital computers are capable of simulating the brain activity of a number of small species, like rats, they consume an increasing amount of energy as the complexity of the animals being simulated rises, which is plainly not feasible. Therefore, it is imperative that computers work like the brains of sophisticated animals without consuming a lot of energy in the future [8–10]. Fortunately, neuromorphic electronics provides a viable solution to the von Neumann bottleneck problem at the hardware level by emulating the functions of biological synapses using artificial electronic devices [11, 12]. The pursuit of technical innovation in the developing field of computer architecture aims to create computers with capacities comparable to the human brain [13, 14].

One of the promising candidates for the next generation memory devices is resistive random-access memory (RRAM), due to its high density, quick switching speed, great scalability, and low power consumption, RRAM, one of the most promising alternatives, has gained increasing attention [1, 15–18]. A typical RRAM device, consisting of a resistive switching (RS) layer sandwiched between two metal electrodes, has a configuration similar to a simple two-terminal capacitor. In past two decades, numerous materials such as Ta_2_O_5_, HfO_2_, ZrO_2_, TiO_2_, ZnO, Al_2_O_3_, CeO_2,_ and among others [2, 12, 19–24], organic materials [25], newly developing perovskites [26] and 2D materials [27, 28] have been studied as RS memory for neuromorphic applications. Because of their excellent compatibility with semiconductor manufacturing technologies, low cost of fabrication, multistate switching, high switching speed, reliability, small cell size, and low power consumption, oxide-based materials have drawn the most attention for use in fabricating RS memory [3, 29, 30]. The performance of memristor devices is highly influenced by the choice of electrode materials. A high work function electrode layer made of Pt or Ni creates a Schottky barrier at the interface with the oxide. On the other hand, the other electrode, known as the ohmic electrode, utilizes a highly reactive oxy-nitride metal such as TiN or TaN to ensure an ohmic contact at the electrode-oxide interface. During the one-time forming process, a conducting oxygen vacancy filament is created in the oxide, connecting the two electrodes and resulting in a low resistance condition when a relatively high voltage is applied to the memristor device. Many studies have reported that oxygen vacancies are produced at the oxide/ohmic electrode (OE) interface during processing and subsequent forming [31].

However, the large-scale commercial deployment of the RRAM device may be hampered by the random nature of conductive filament production in oxide-based memory devices during the switching operation. To overcome this issue, a few successful strategies have been developed to limit the excessive growth of conductive filaments based on oxygen vacancies in the switching region of the memristor. Numerous process optimizations, including metal doping, integrating reactive metal layers, multilayer architectures, and metal nanocrystal incorporation, were employed to address the variability difficulties of RRAM memory devices [32–35]. The strategies mentioned above have been the subject of numerous research articles. The challenges with repeatability and uniformity during switching cycles of the memory devices continue to be the biggest challenge for approaches. However, there are not many studies on RRAM devices constructed mixture of two oxide elements. Kim et al. [36] examined the RS characteristics in an alloy memory system based on HfTiO_x_. Lee et al. [37] demonstrated fundamental RS properties and achieved multilevel switching behavior by applying the DC sweep and pulses. Wang et al. [38] obtained analog switching behavior and synaptic function by interface-engineering in HfO_2_-based RRAM through O_3_ pulse treatment. Roy et al. [39], successfully demonstrated the reliability of HfO_2_-based RRAM devices for synaptic simulation by incorporating Al doping and conducting postdeposition annealing (PDA). Biswas et al. [40] have successfully improved the cycle-to-cycle and cell-to-cell uniformity of the HfO_x_-based memristor, as well as the switching resistance ratio, which now exceeds 10^3. This was achieved by incorporating an interfacial AlO_y_ layer, which plays a crucial role in controlling the formation and rupture of the conductive filament. Zhu et al. [41] have developed an interface engineering methodology that involves introducing a thin layer of NiO_x_ into a sandwiched Al/TaO_x_/ITO resistive switching device, where the NiOx/TaOx interface barrier is able to confine the formation and rupture of filaments throughout the entire bulk structure under critical bias setups. An mixture of two oxide-based memristor that may be used in memory computing is greatly desired because it has dependable memristor performance and a wealth of memory logic functionalities. As was previously said, atomic layer deposition (ALD) is regarded as one of the most widely used techniques for depositing ultrathin films because of its special benefits, which include great homogeneity, exact atomic-scale thickness control, and low deposition temperature. As a result, constructing an ideal artificial synapse, which is still a challenge, depends on stable oxide-based memristor that are simple to replicate.

Synapses between neurons in biological synapses transmit and process action potential signals, which results in complex neurological activity. Synaptic plasticity, which is further divided into short-term plasticity (STP) and long-term plasticity (LTP), allows for efficient modification of the strength of connections between neurons under purposeful stimulation. In the past, scientists have investigated and created artificial synaptic devices having both STP and LTP functionalities, such as metal oxide semiconductors [42] chalcogenides [43], nanoscale two-terminal memristors, and three terminal synaptic transistors [44, 45]. The physical mechanism of the artificial synaptic device can be attributed to the controlled movement of ions in response to electrical stimulation, which leads to additional nonlinear conductance changes accompanied by the activation of post-synaptic currents (PSC) [46]. Moreover, researchers have explored various types of plasticity in artificial synaptic devices, including spike-time dependent plasticity (STDP) and paired-pulse facilitated plasticity (PPF), among others. Further research is being conducted in this area to enhance the capabilities of these devices and make them more similar to biological synapses [47]. A predictable and repeatable configuration of resistance states for storing synaptic weights in artificial neural networks (ANNs) is a significant difficulty for memristor-based devices in neuromorphic systems. High durability and consistency of the device properties over numerous switching cycles are necessary [48]. In general, the type of weight update used by any neuromorphic system affects the needs for the memory device parameters. For instance, the spike-timing dependent plasticity (STDP) rule necessitates a time-dependent, nonlinear resistance change and is most significant for spiking neural networks (SNN) [49]. As opposed to this, traditional ANNs (such as deep neural networks or convolutional neural networks) need a linear resistance change and as many stable resistance values as they can handle [50]. Although many areas of neuromorphic computing have shorter memory length requirements than nonvolatile memories, concise state retention periods prevent the devices from having many applications in neuromorphic computing [51, 52].

In this paper, an a-HfSiO_x_ based memristor was fabricated using ALD to investigate its electrical and biological properties for multilevel switching and neuromorphic computing systems. The Pt/a-HfSiO_x_/TaN memristor demonstrated typical bipolar RS behavior and improved retention performance (10^4^ s), cycle stability (1000 cycles), and voltage distribution. Its multilevel capability was demonstrated by adjusting the current compliance (CC) and reset-stop voltage. The memristor demonstrated the ability to replicate numerous synaptic properties, such as paired-pulse facilitation (PPF), potentiation, depression, and spiking-rate-dependent plasticity (SRTDP). The accuracy of the memristor in pattern recognition was assessed through simulation and utilizing the modified National Institute of Standards and Technology (MNIST) dataset. Analysis of the crystal structure and chemical distribution of the a-HfSiO_x_/TaN layers was conducted through X-ray diffraction (XRD) and X-ray photoelectron spectroscopy (XPS), respectively. These findings demonstrate the superior RS performance of a-HfSiO_x_ for multilevel switching and neuromorphic computing systems.

## Experimental procedure

### a-HfSiO_x_-based memristor device fabrication

The following two terminal memristor was designed with a Pt/HfSiO_x_/TaN stack arrangement. First, the Si-substrate (SiO_2_/Si) was cleaned by ultrasonication for 5 min in successive baths of acetone and deionized (DI) water. Then, a bottom electrode was created by depositing tantalum-oxy nitride (TaN) on a Si/SiO_2_ substrate using direct current (DC) magnetron reactive sputtering (BE). Using 250 W of sputtering power in a pure argon environment at ambient temperature, the TaN film was created (RT). The hafnium-silicon-oxide (HfSiO_x_) layer was then applied using the ALD system (CN-1). The combination of Hf/Si precursor and oxygen source with TEMAHf/DIPAS and O_3_ flow, along with N_2_ as the purging gas, was used in the deposition process. Five layers with a 1:3 ALD cycle ratio (SiO_2_:HfO_2_) were prepared with a thickness of ~ 5 nm, deposited by 60 cycles. The HfSiO_x_ film was produced using a TEMAHf/DIPAS pulse of 0.8 s, a 15 s N_2_ purge, an O_3_ pulse of 0.5 s, and another 15 s N_2_ purge. The stage temperature was set to 400 °C. A circular metal shadow mask was formed on the top surface of the a-HfSiO_x/_TaN substrate to determine the memristor cell size. Each cell had a diameter of 100 μm. Finally, a 100 nm Pt film was deposited on the by using an e-beam evaporator (ULVAC, FF-EB20), used as the top electrode (Pt) in the argon atmosphere at RT.

### Electrical and biological characterizations

After construction of the Pt/a-HfSiO_x_/TaN memristor, Keithley 4200-SCS and 4225-PMU semiconductor parameter analyzers equipped with a probe station were used to assess current-voltage (I-V), cycle endurance, data retention, and biological properties. Bias was always applied to the TE while maintaining the BE grounded during all measurements.

### Materials characterization

Utilizing the x-ray diffractometer (XRD) Bruker D8 Advance with Cu K_α_ radiation (λ = 1.5414 Å) spanning from 20° to 80° at a step size of 0.05° min^− 1^, the crystal structure of the HfSiO_x_/TaN film was investigated. The sample was prepared using a focused ion beam (FIB) for TEM examination prior to the transmission electron microscope (TEM) measurement. Field emission TEM was used to study the Pt/a-HfSiO_x_/TaN memristor device in cross-section (Talos). Ex situ x-ray photoelectron spectroscopy (XPS, ULVAC-PHI/X-TOOL), using an Al K_α_ (1486.6 eV) rastering over a 200 μm $\times$200 µm area at 12 kV, was used to analyze the chemical composition of the HfSiOx/TaN stack. The peak of the C 1s spectrum was used as the calibration point for all XPS spectrum curves, with a comprehensive scan pass energy of 200 eV. The XPS and TEM analyses were carried out following the schematic diagram illustrated in Additional file 1: Fig. S1.

## Results and discussion

**Fig. 1:**
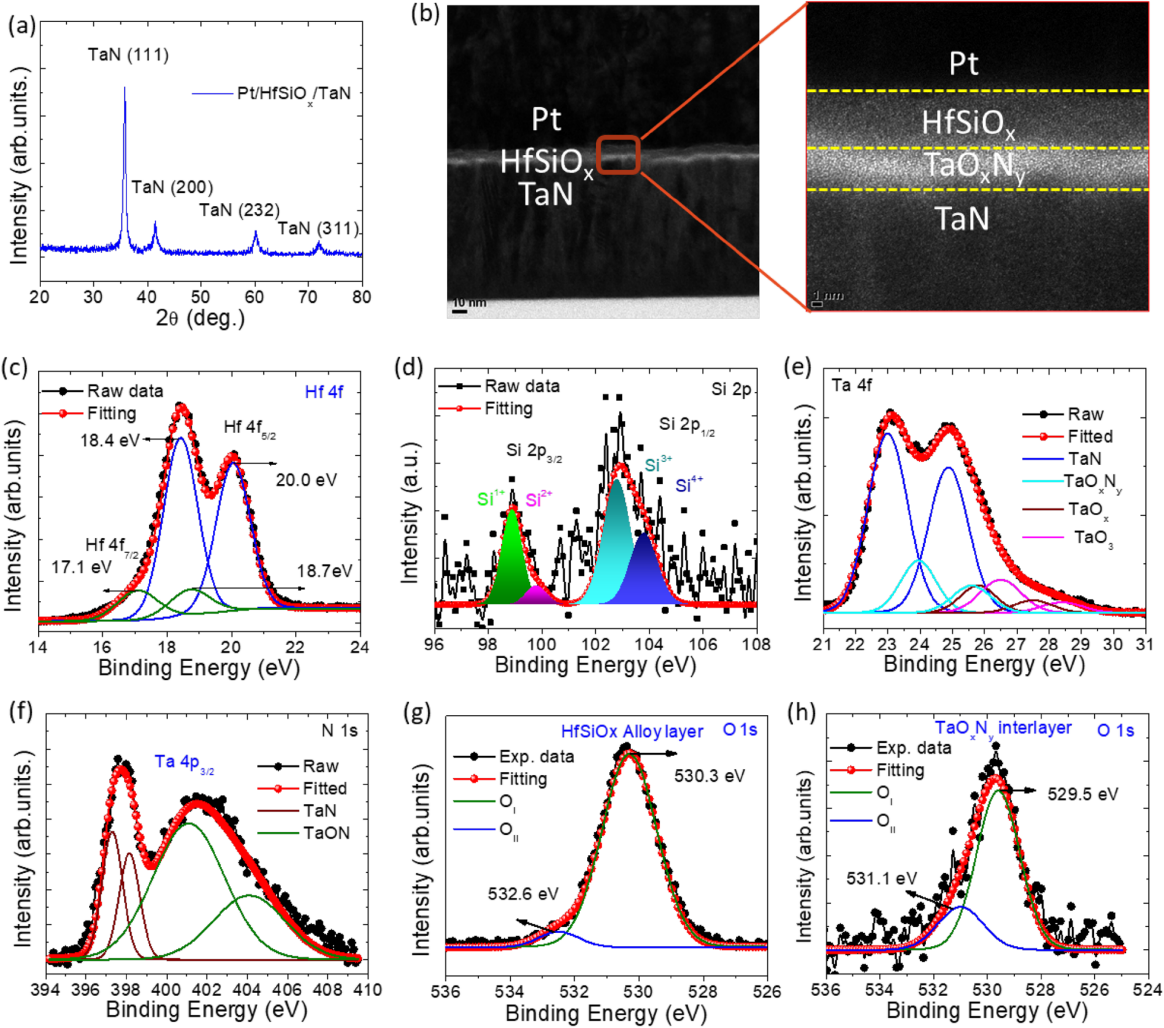
**a **XRD pattern of the TaN substrate and a-HfSiO_x_ thin film. **b** The low magnification and magnified HRTEM image of the Pt/a-HfSiO_x_/TaN memristor. The XPS spectra of **c** Hf 4f, **d** Si 2p, **e** Ta 4 **f**, (Materials characterizations.) N 1s, and O 1s spectra of **g** a-HfSiO_x_ layer and **h** TaO_x_N_y_ interface layer in Pt/a-HfSiO_x_/TaN memristor

Figure 1(a) illustrates the XRD pattern of the HfSiO_x_/TaN substrate. The XRD results indicated that all peaks are related to the TaN electrode, with no prominent diffraction peaks for an HfSiO_x_ crystalline phase. As a result, the a-HfSiO_x_ insulating layer produced by ALD growth was really amorphous, assuring a smooth thin-film morphology and likely aiding in the memristor stable analog switching properties. The XRD results suggested that the a-HfSiO_x_ film has an amorphous phase. Figure 1(b) shows the cross-sectional and high-resolution TEM image of the Pt/a-HfSiO_x_/TaN memristor after direct current (DC) sweeping electrical measurement. It is evident that the bottom HfSiO_x_/TaN interface generated an interface layer, which resulted in a chemical reaction between the high oxygen affinity tantalum-oxynitride TaN BE and the a-HfSiO_x_ insulating layer, similar to our earlier findings [53, 54]. The HRTEM image further demonstrates the amorphous nature of the HfSiO_x_ insulating layer. Additionally, the switching and interface layers must have thicknesses of no more than 5 nm and 2 nm, respectively. The structures of the a-HfSiO_x_ film are well congruent between the XRD and TEM analyses. Additional file 1: Fig. 2S(a) illustrates a depth profile of the chemical composition over etching time. As expected, the atomic concentration of Pt decreases significantly, while the concentrations of O, Hf, and Si increase as sputtering reaches the TaON/TaN interface. After 10 s of etching time, inter-diffusion of various species, such as Ta, N, and O, becomes evident, confirming the existence of a TaON interfacial layer between the TaN bottom electrode and the adjacent HfSiOx film. Additional file 1: Fig. 2S(b) and 2S(c) show the EDS image and atomic percentages of Pt, Si, Hf, O, N, and TaN in the Pt/HfSiO_x_/TaN memristor device. The interfacial layer between the TaN bottom electrode and the HfSiO_x_ resistive switching layer is crucial in improving the performance of Pt/a-HfSiO_x_/TaN RRAM devices. However, the TaN electrode causes redox reactions at the oxide and oxide/metal interface due to its high oxygen affinity and low oxide formation energy [55, 56]. These redox reactions affect the formation and rupture of conductive filaments, resulting in unintended phenomena in the resistive switching behavior. Additionally, TaN acts as a barrier that controls the injection and extraction of charge carriers, while also trapping and releasing charge carriers, thereby affecting the device’s resistance state [57, 58]. When TaN contacts HfSiO_x_, it leads to the formation of a non-stoichiometric TaO_x_N_y_ interlayer with a high content of oxygen vacancies near the interface, which increases the available free space for oxygen ion migration and weakens the local oxygen-cation bonds [57, 59].

XPS investigations were conducted to comprehend the composition and chemical bonding states of the Pt/a-HfSiO_x_/TaN memristor. It was done to normalize the core-level spectra of Hf 4f, Si 2p, Ta 4f, N 1s, and O 1 s, respectively. With the use of Gaussian-Lorentzian functions, all spectra are simulated. As illustrated in Fig. 1(c), the Hf 4f spectra can be divided into two groups of peaks. High-intensity Hf 4f_7/2_ and Hf 4f_5/2_ are attributed to Hf-O bonding from HfO_2_ (Hf^4+^) at energies of 18.4 eV and 20.0 eV, respectively, with the spin-orbit splitting of 1.6 eV [60]. The lower binding energies of 18.1 eV and 18.7 eV, which are the weaker spin-orbit doublet peaks, may be due to the oxygen deficiency of Hf^n+^–O (n < 4), which comes from HfSiOx. These binding energies are consistent with previously published data [61]. Figure 1(d) shows the XPS Si2p core-level spectra. The XPS showed that the HfSiOx film had a substantial amount of Si-suboxides (Si^+^, Si^2+^, and Si^3+^), in addition to regular SiO_2_ (Si^4+^) [62, 63]. The Ta 4f spectra, shown in Fig. 1(e), can be separated into four pairs of Ta 4f_7/2_ – Ta 4f_5/2_ doublet peaks. The first doublet has peaks at 23 and 24.8 eV, representing the Ta-N bond [64]. In the second doublet, peaks at 23.9 and 25.7 eV are related to TaO_x_N_y_ [43]. The third doublet has peaks at 25.8 and 27.5 eV, related to TaO_x_ [65] and resulting from the Ta 4f_7/2_ and Ta 4f_5/3_ electronic states. Finally, the fourth doublet, with peaks at 26.6 eV and 28.4 eV, represents the Ta 4f_7/2_ and Ta 4f_5/2_ electronic states and is associated with TaO_3 − x_. The N 1s core-level spectra, which are displayed in Fig. 1(f), further supported the establishment of the TaO_x_N_y_ interface layer. While peaks at 401. 1 and 404.0 eV are indicative of TaO_x_N_y_ [66], N 1 s peaks at 397.2 and 398.1 eV have formed from the TaN surface. This proves that the oxy-nitride compounds contain Ta-O bonds and Ta-N bonds [64–66]. According to the literature, tantalum nitrides can easily be converted into tantalum oxy-nitride (TaO_x_N_y_, TaON) [67]. It is reasonable to assume that a TaON interfacial layer has also been generated between the HfSiO_x_ and TaN films when TaN is used as the bottom electrode, as confirmed by the TEM as mentioned above data presented in Fig. 1(b). We think that during the high-temperature ALD deposited of the a-HfSiO_x_ insulating layer, the oxygen plasma partially oxidized the surface of the TaN film and created the TaO_x_N_y_ interface layer [68].

The TaO_x_N_y_ interface layer and the HfSiO_x_ insulating layer, O 1s XPS spectra were de-convoluted into two peaks using Gaussian functions, as shown in Fig. 1(g,h). The terms “oxygen ions” and “oxygen vacancy” refer to oxygen ions (O_I_) and non-lattice oxygen (O_II_), respectively. The area percentage of each peak can be used to determine the relative oxygen vacancy concentration in the bottom TaO_x_N_y_ interface layer and the a-HfSiO_x_ insulating layer [69, 70]. In the bottom TaO_x_N_y_ interface layer and the a-HfSiO_x_ insulating layer, the computed percentage concentration of oxygen vacancies is around 10% and 30%, respectively. According to our earlier research [71, 72], the primary peak of O 1s in the HfSiO_x_ insulating layer is 530.3 eV, which is 0.8 eV higher than the TaO_x_N_y_ interface layer. This indicates the formation of a sub-oxidized layer. As a result, interface chemical interactions lead to the creation of TaO_x_N_y_ interface layers, which in turn causes the next a-HfSiO_x_ insulating layer to produce more oxygen vacancies. The EDS depth profile analysis (Additional file 1: Fig. S2(a)) demonstrates that the oxygen content within the TaO_x_N_y_ interlayer consistently decreases along a profile extending from a-HfSiO_x_ to TaN. Consequently, this process leads to the diffusion of oxygen ions along the same profile. The elevated concentration of oxygen vacancies near the interface boosts the availability of free space for oxygen ions to migrate and reduces the local oxygen-cation bonds, thereby facilitating the migration of oxygen ions [73].

**Fig. 2:**
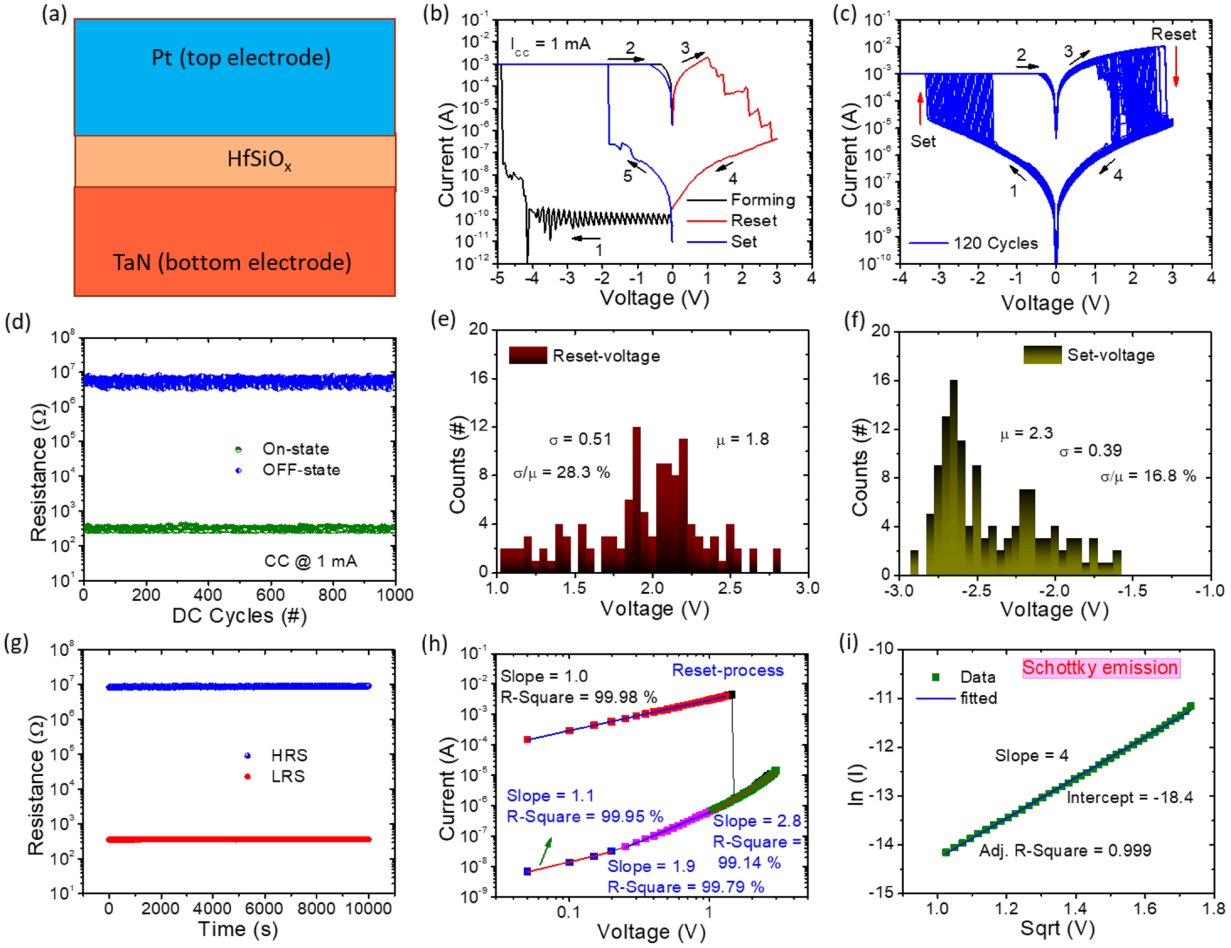
**a** A Pt/HfSiOx/TaN memristor device image. **b** Typical current–voltage (I–V) curves showing the forming, reset and set operations, **c** Repeated DC switching cycles totaling 120 throughout set and reset procedures. (**d**) Endurance performance over 1000 cycles. 200 cycles of the **e**,**f** set and reset voltages were distributed statistically. **g** Properties of retention at RT **h** Log-log scale was used to fit the I–V curve. **i** Schottky emission-fitted High HRS high field area. Inset of Fig. **h**,**i**, indicate that the R-square values are greater than 99.9%

The RS characteristics of the produced Pt/HfSiO_x_/TaN sandwiched structure were examined to assess its potential for high-density data storage applications. The Pt/a-HfSiO_x_/TaN memristor is shown schematically in Fig. 2(a), with the top layer being a Pt (100 μm in diameter), the middle switching layer being a thin film constructed of a-HfSiO_x_ insulating, and the bottom layer being a TaN electrode for electrical measurements. Usually, a forming process is necessary to initiate the fresh cell into the effective switching state, as seen in Fig. 2, before the memristor film exhibits any switching characteristics. As shown in Fig. 2(b), during the negative voltage sweep from 0 to 5.0 V and back to 0 V, the high resistance state (HRS) sharply drops to the low resistance state (LRS) at around 4.6 V, a phenomenon known as the forming process. In contrast, the memristor was reset by switching back to HRS at roughly + 1.0 V. A negative voltage sweep was then used to execute a set process. It should be noted that the forming voltage (about 4.6 V) is higher than the SET voltage (about 1.5 V), and the initial leakage current is considerably lower than the HRS leakage current following forming. The memristor device current compliance (CC) was fixed at 1 mA during the forming and set operations to prevent hard breakdown. On the other hand, the reset process had no current cap. The stability test results for 120 consecutive sweeping I-V curves are shown in Fig. 2(c). The device demonstrates relatively good cycle stability and repeatability, as evidenced by the tendency of the I-V curves to remain constant with increasing sweeping cycles. The endurance performance over 1000 cycles is shown in Fig. 2(d). The LRS and HRS values were extracted at a read voltage of 0.2 V. The proposed memristor has reliable reproducibility, as evidenced by the fact that the device was kept running with a sizable memory window (> 10^4^). Additionally, as shown in Fig. 2(e,f), statistical analyses of the set and reset voltages of the memristor are displayed in the histograms. The Gaussian function is used to fit the V_set_ and V_reset_ distributions. It should be noted that the statistical count only represents the V_set_ and V_reset_ within a certain range. The V_set_ and V_reset_ have a concentrated distribution that is primarily found at 2.3 and 1.8 V, respectively. The set and reset voltages have coefficients of variation (σ/µ), standard deviations (σ), and averages (µ) that are 16.8% and 28.3%, respectively. The homogeneity of the operation voltage serves as the foundation for the memristor device practical application. Figure 2(g) displays the LRS and HRS’s retention characteristics at RT. Under a steady 0.2 V reading bias voltage provided for 10^4^ s, the HRS and LRS resistance remains unchanged. All of the findings above show that the built oxide-based memristor maintained a high level of stability, displaying high-reliability RS with no misreading of resistance states or switching failure during the test. The RS characteristics of five memristor devices were evaluated by performing 100 consecutive I-V sweeping cycles and assessing their endurance performance. This evaluation is illustrated in Additional file 1: Fig. S3 (a–e) and Fig. S4 (a–e). These cycles were repeated to assess reliability and reproducibility of each memristive device. Furthermore, three devices were subjected to reliability and reproducibility tests at various constant current (CC) levels, namely 3 mA, 5 mA, and 10 mA. The results, presented in Additional file 1: Fig. S5(a–f), indicate that there are no notable differences in performance. This confirms the dependable and consistent RS characteristics of the Pt/a-HfSiO_x_/TaN memristor.

To demonstrate the current transport conduction and switching mechanism of the memristor. The I–V characteristics curve is re-plotted in Fig. 2(h) on a log-log scale. The IV curve in the HRS is separated into three zones. The slope of the fitting line is roughly 1.1 in the low voltage region between 0 and 0.2 V, demonstrating the Ohmic conduction (I ∝ V) brought on by thermally produced charge carriers. The slope of the fitted line increases to 1.9 as the applied voltage rises (0.25 to 1.0 V), indicating trap-unfilled conduction behavior (I ∝ V^2^). The slope is 2.8 in the area of the high field (1.05 to 3.0 V), which is consistent with trap-filled conduction behavior (I ∝ V^n^, n > 2). The memristor will switch from HRS to LRS when the external voltage hits 3 V. The I-V curve is once again in perfect accord with Ohmic conduction in the LRS. The I-V curve is nicely fitted with the Schottky emission conduction mechanism with a slope of 4.0, as shown in Fig. 2(i), after a study of the high voltage area of the HRS. The measured linear relationship of vs. with voltage in the region of 1.05 to 3.0 V indicates Schottky emission is primarily responsible for the current conduction mechanism in this range [74, 75].

1 $$J={A}^{*}{T}^{2}exp\left[\frac{-q\left({\varphi }_{b}-\sqrt{qE/4\pi {\epsilon }_{o}{\epsilon }_{r}}\right)}{{k}_{b}T}\right]$$

where ${A}^{*}$is the Richardson constant, J refers to current density, ${\varphi }_{b}$, refer to Schottky barrier height, q refers to electronic charge, E refers to the electric field, ${\epsilon }_{0}$ refer to free-space permittivity, ${\epsilon }_{r}$ refer to relative permittivity, ${k}_{b}$ refer to the Boltzmann constant, and T is the absolute temperature (300 K). The HfSiOx layer at HRS is typically protected by dielectric films that agree with the TaO_x_N_y_ interface layer and use the Schottky emission model to describe the conduction mechanism. To create TaO_x_N_y_, the active TaN BE absorbs some oxygen ions from the HfSiO_x_ insulating layer, enhancing the oxygen vacancies close to the interface.

The negatively charged oxygen vacancies travel toward the BE as the memristor is negatively biased and are finally blocked by the HfSiO_x_, generating an oxygen vacancy conductive filament across the HfSiOx insulating layer. The concentration gradient of oxygen ions across the a-HfSiO_x_ layer results in a significantly higher number of oxygen vacancies near the TaN electrode than that near the HfSiOx insulating layer, leading to a cone-shaped conductive filament [76, 77]. The a-HfSiO_x_ layer acts as a “wall” to prevent the accumulation of oxygen vacancies at the cathode and maintains the proper shape of the conic filament. This layer has a higher vacancy formation energy than amorphous HfSiO_x_. The conductive channel is modeled as an Ohmic resistor, as shown in the fitting result in Fig. 2(h). On the other hand, the conductive filament is ruptured when we supply a positive voltage to reset the memristor by removing the oxygen vacancies from the cone tip. This operation uses relatively high energy.

**Fig. 3:**
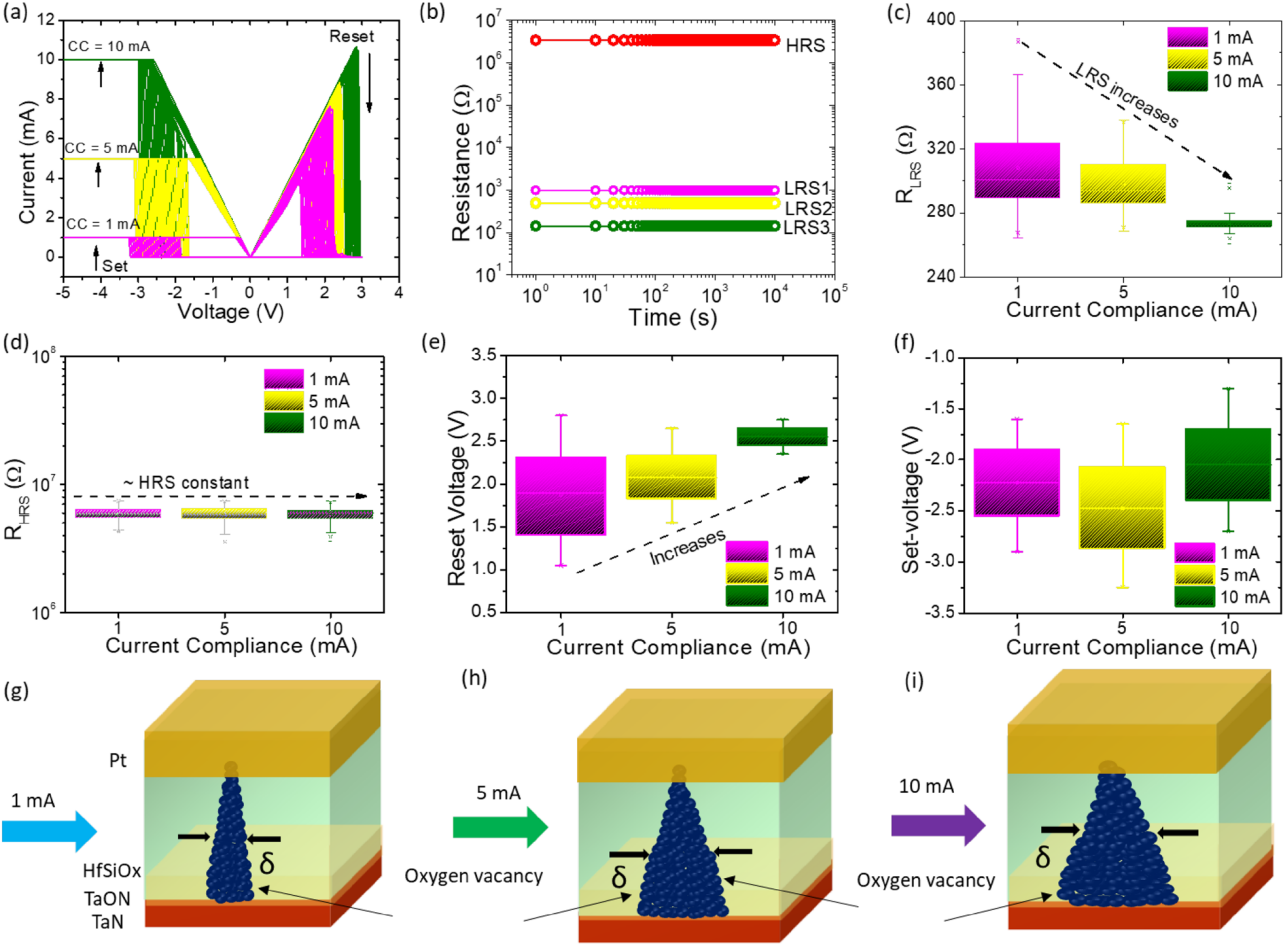
Characteristics of multilevel switching: **a** 50 consecutive switching cycles, **b** retention characteristics **c** LRS, **d** HRS, **e** RESET-voltage, **f** SET-voltage under three distinct CCs (1 mA, 5 mA, and 10 mA). Multilevel switching process illustrated schematically for CC values of, **g** 1 mA, **h** 5 mA and **i** 10 mA, respectively

According to published research, the CC during the set process is a critical element of memristor device features for high-density data storage with the economy. CC control is used to implement multilevel programming during the set process. Figure 3(a) displays the switching I–V curve of the memristor device under a range of CC of 1 mA, 5 mA, and 10 mA. Figure 3(b) reveals the retention performance for various CCs. Limiting CCs, also known as LRS1, LRS2, and HRS, of 1 mA, 5 mA, and 10 mA yield three LRSs and one HRS. In this scenario, four states can maintain a reading voltage of less than 0.2 V at RT for 10^4^ s, proving the memristor outstanding retention performance. The impact of the LRS and HRS on the CC is depicted in Fig. 3(c–d). As can be seen, the memristor LRS can be discriminated against at different CC levels, but its HRS is unaffected by CC. The influence of CC on set and reset voltage is seen in Fig. 3(e–f). It was found that increasing CC led to changes in the reset and set voltage, both related to the diameter of the conductive filaments, respectively. As compared to set-voltage, reset voltage increases with CC. The increasing trend in reset voltages suggests that a higher CC limitation results in the formation of more “robust” filaments that subsequently need larger reset voltage to rupture the filament [78].

Figure 3(g–i) depicts the schematic switching mechanism of the memristor device, which can be described as a gradual increase in conductive filament size brought on by raising CC during the set process [20, 79, 80]. Following past findings, the length of the conducting path grows as CC rises. The increased density of point defects, which through repeated switching cycles results in conductive filaments, is what causes this [12]. Thus, the TaO_x_N_y_ interface layer serves as a storage space for oxygen ions as the resistance of LRS falls, resulting in discrete resistance states at various CC. The filaments size starts to increase as the CC increases. By altering the diameter of the conductive filament, one can get various resistance states or multilevel switching operations.

**Fig. 4:**
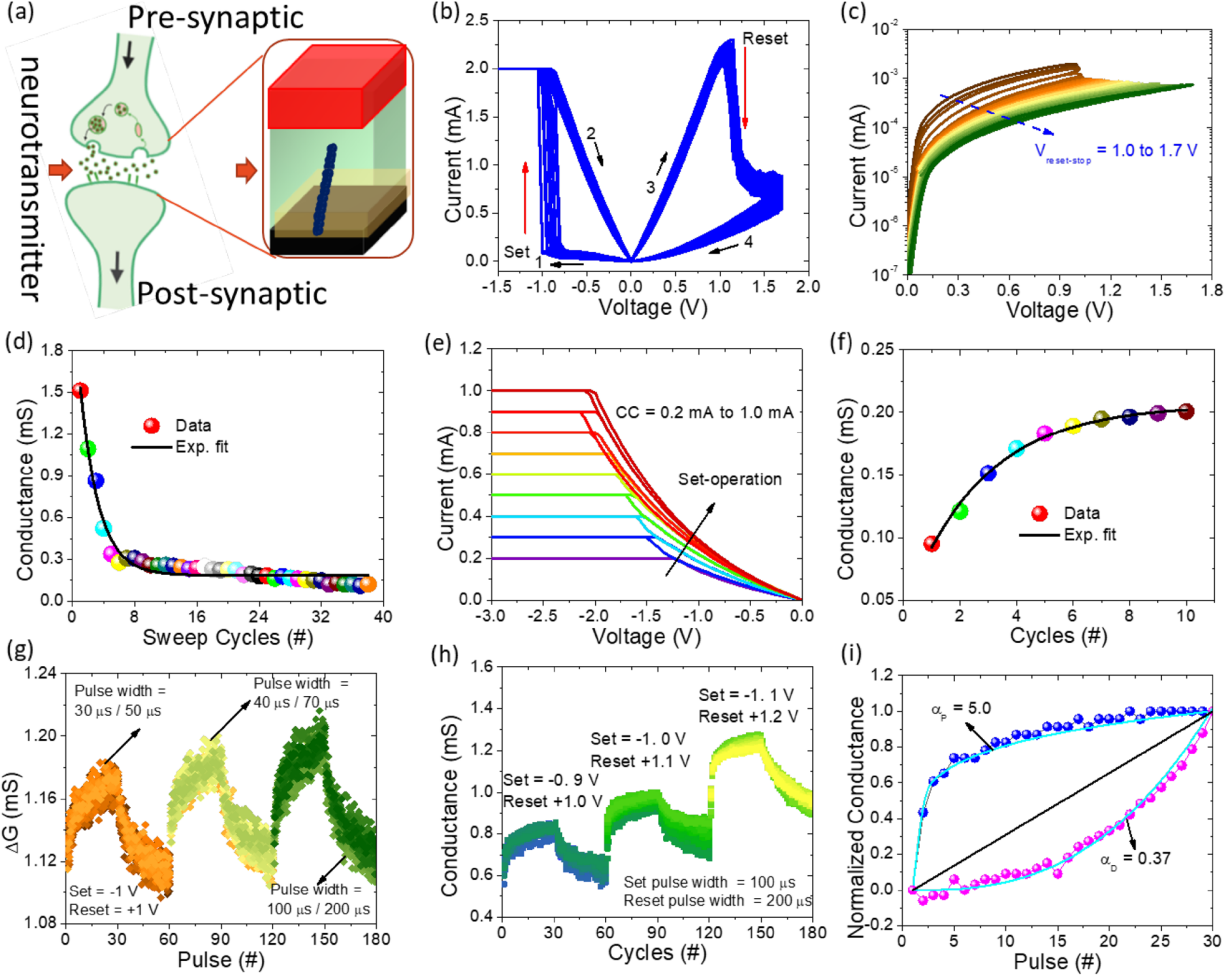
**a** A simplified diagram of a biological synapses based on Pt/a-HfSiO_x_/TaN memristor with two terminal structures. **b** I–V characteristics were replicated for 100 cycles before implement of biological synapses. **c** Gradual reset method using positive sweeping voltages that are increased (+ 1.0 V to + 1.7 V, step value = 0.01 V). **d** Variations in conductance levels during a string of successful sweeps. **e** Gradual set with CC increased (0.2 mA to 2 mA with a 0.01 mA increment) while the set operation is in progress. **f** Modifications to conductance levels by CC control during set-process. The 30 negative pulses followed by 30 positive pulses, each with variable **g **pulse width and **h** pulse amplitude features of potentiation and depression. **i** A Pt/a-HfSiO_x_/TaN memristor with a high pulse amplitude of − 1.1 V/100 µs for potentiation and + 1.2 V/200 µs for depression produced synaptic weight update nonlinearity

The schematic representation of the synapse and memristor structure, which primarily consists of pre-synaptic neurons, neurotransmitters, and postsynaptic neurons, is shown in Fig. 4(a). A pre-synaptic neuron and a postsynaptic neuron are separated by a tiny space known as the synapse. A biological synapse is comparable to a two-terminal memristor device. Pt TE and TaN BE similarly mimic pre- and postsynaptic neurons. The pre-synaptic spike onto the pre-synapse is analogous to the applied electric pulse on the TE, which stimulates ion migration via the a-HfSiO_x_ switching layer and causes a change in conductance. Since varying synaptic strength impacts synaptic plasticity, the a-HfSiO_x_ insulating layer mimics the synaptic cleft, allowing neurotransmitters to pass from pre- to post-neuron [81]. Basically, synaptic plasticity, the mechanism behind signal transmission between two neurons, is a change in synaptic weight in response to environmental inputs.

Consequently, the synaptic connection becomes more robust, which temporarily increases synaptic weight. The postsynaptic potential or current indicates the synaptic weight [82]. Our memristor can serve as a demonstration of such an analogy. The memristor 100 I–V characteristics curves were measured before testing the analog switching behavior, as can be seen in Fig. 4(b). After that, the conductance state can be gradually adjusted by adjusting set CC and stop-voltage reset voltage, respectively. As shown in Fig. 4(c), the positive sweeping voltages, which are varied from 1.0 to 1.7 V with 0.001 V decrements, are controlled to stabilize multilevel states during the reset process. The conductance vs. reset-voltage plot is shown in Fig. 4(d). By stopping the reset voltage, it can be seen that conductance steadily decreases. This pattern resembles biological synapses depressive characteristics. Similar to how numerous states can be successfully attained by adjusting CC in the set process (see Fig. 4(e)). The CC is raised from 0.2 to 1.0 mA with an increment of 0.1 mA to achieve multilayer states, but no reset process is necessary. The conductance vs. CC plot, which resembles the potentiation properties of biological synapses, is shown in Fig. 4(f). These results show the superior analog conductance switching capability of the Pt/HfSiO_x_/TaN memristor, which satisfies design requirements and suggests a high potential for neuromorphic computing applications.

Potentiation and depression properties are examined in the Pt/a-HfSiO_x_/TaN memristor utilizing various pulse widths and stimulation amplitudes to confirm the memristor’s adjustable conductance. A well-controlled analog state is depicted in Fig. 4(g) by the memristor potentiation and depression characteristics under 30 consecutively negative pulses (− 1.0 V) and 30 consecutively positive pulses (+ 1.0 V), with different pulse widths of 30/50 µs, 50/70 µs, and 100/200 µs, respectively. The same is true for Fig. 4(h), which shows the potentiation and depression characteristics under identical negative (– 0.9 V/100 µs, − 1.0 V/100 µs, − 1.1 V/100 µs s) and positive (+ 1.0 V/200 µs, + 1.1 V/100 µs, and + 1.2 V/200 µs) pulse trains with the same interval. Under various pulse amplitudes and widths, the 20 cycles were repeated for each set of potentiation and depression cycles, indicating a controllable conductance change in the Pt/HfSiO_x_/TaN memristor that is applicable to synaptic characteristics [83, 84]. By adjusting the pulse width and amplitude, it can be shown that a progressive rise and fall in conductance was noticed as a result of the formation and rapture of nanofilament filaments between the a-HfSiO_x_ insulating layer and TaN BE [85]. After 20 programming cycles, there are 60 levels of conductance state that can be acquired for synaptic weight storage, as shown in Fig. 4(i). The change in conductance for potentiation ${(G}_{P})$ and depression ${(G}_{D})$ with the number of pulses $\left(P\right)$ can be represented by the following equations, which describe the linearity of weight update during the potentiation- and depression-process [86, 87].

2 $${G}_{P}=B \left(1-{e}^{\left(\frac{- p}{A}\right)}\right)+ {G}_{min}$$

3 $${G}_{D}=-B \left(1-{e}^{\left(\frac{P- {P}_{max}}{A}\right)}\right)+ {G}_{max}$$

4 $$B= \frac{{G}_{max}-{G}_{min}}{1-{e}^{\frac{-{P}_{max}}{A}}}$$

5 $$= \frac{1.08}{A+0.127}$$

where, ${G}_{max}$ is the maximum conductance, ${G}_{min}$ refer to minimum conductance and ${P}_{max}$ denotes the maximum number of pulses required to tune the memristor from minimum to maximum conductance state. The parameter A determines the nonlinearity behavior of weight updates, while B is a function of A, and is a parameter that characterizes the degree of nonlinearity. The results of the fitting are shown in Fig. 4(i), where the nonlinearity parameter of the Pt/a-HfSiO_x_/TaN memristor is, respectively, 5.0 for potentiation and 0.37 for depression.

**Fig. 5:**
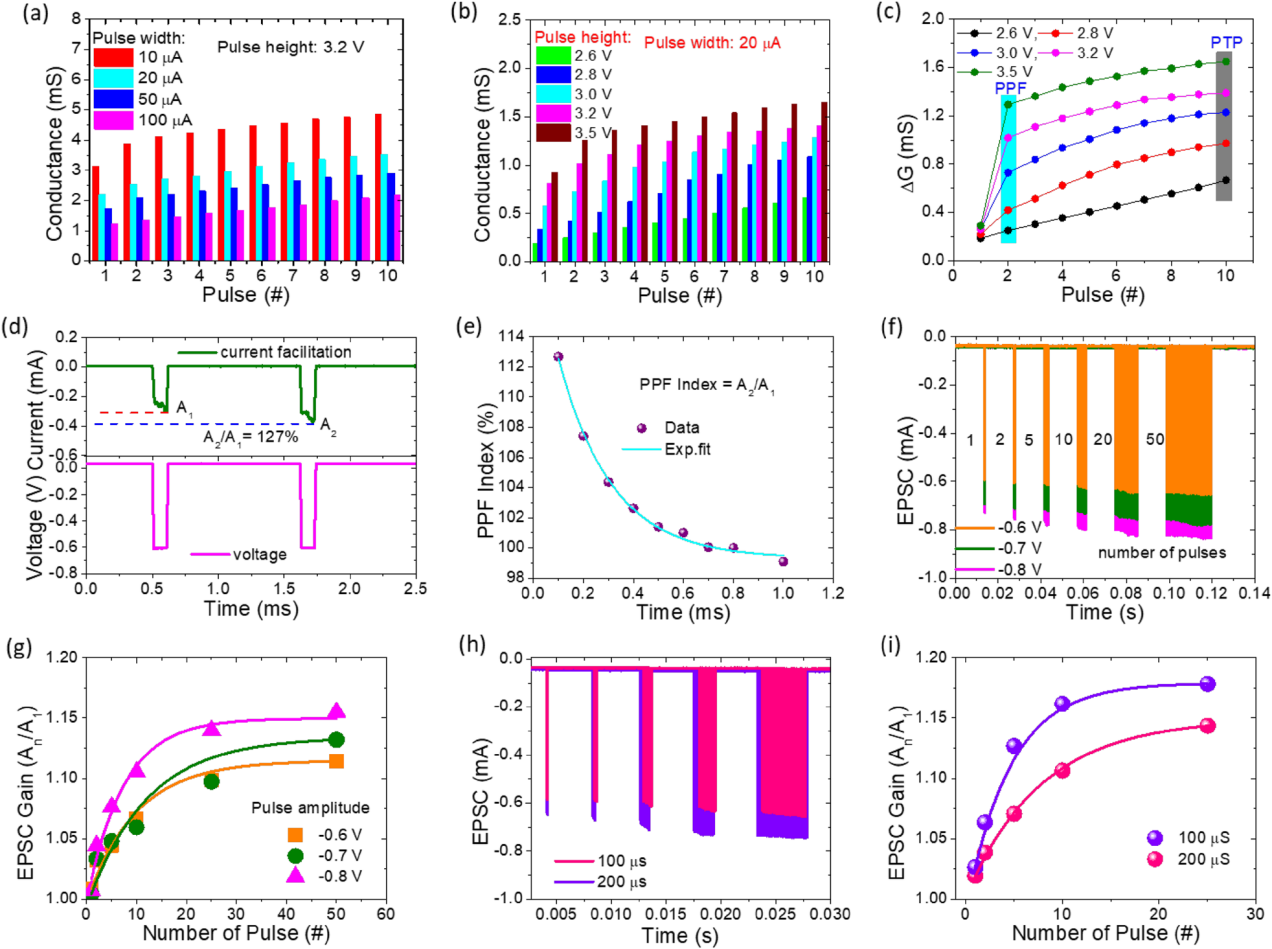
Short-term synaptic plasticity of the electronic synapse: Variations in the absolute current values (or synaptic weight) with respect to potentiating pulse number for different **a** pulse intervals, **b** pulse amplitudes. **c** Mean changes (ΔI) of current during cycles of 10 pulses at different pulse amplitudes. **d** PPF behaviors of Pt/HfSiOx/TaN memristor with postsynaptic EPSCs triggered by a pair of presynaptic spikes, with 0.1 ms interval times. **e** PPF index as a function of the inter-spike time interval between two pulses. The light blue curve represents the exponential fit. **f** Modulation of the EPSC change with different pulse voltage amplitudes from ‒ 0.6 V to ‒ 0.8 V and spike numbers ranging from 1 to 50, respectively. **g** EPSC gain in relation to the quantity of pulses. **h** EPSC responses at various pulse widths of 100 µs and 200 µs while increasing the number of spikes from 1 to 25 at a constant pulse amplitude of ‒ 0.7 V. **i ** EPSC gain as a function of various pulse counts

By altering a synaptic weight, synaptic plasticity is seen as a learning and memory function in neuromorphic systems [88]. Spike-rate-dependent plasticity (SRDP) is a fundamental aspect of synaptic plasticity [85]. Changing the inter-pulse interval changed the stimulation rate. The conductance modulation characteristics of the memristor device under various pulse intervals are shown in Fig. 5(a). We looked at ten identical pulses with fixed pulse peak (–3.2 V), fixed pulse width (10 µs s), and fixed pulse intervals (10 µs, 20 µs, 50 µs, and 100 µs). The relationship between the pulse interval and conductance modulation of the memristor is clearly evident. Shorter inter-pulse intervals lead to a rapid increase in memristor conductance, while longer inter-pulse intervals result in slower conductance updates. This demonstrates the successful replication of the spiking-rate-dependent plasticity (SRDP) features of biological synapses, indicating a strong dependence of conductance on spike firing rate. Moreover, the memristor’s conductance modulation features were studied for pulse amplitude dependence. Five different pulse trains were applied, each containing 10 pulse stimulations with varying pulse heights (−2.6 V, −2.8 V, −3.0 V, −3.2 V, and − 3.5 V) but the same pulse width (10 µs) and pulse interval (10 s), revealing the spike amplitude-dependent weight modulation characteristics. Figure 5(b) shows the memristor conductance response. Similar to SRDP behavior, the memristor apparently relied on the pulse height on conductance modulation. This demonstrates that a spike amplitude-dependent plasticity process exists and that the pulse stimulations’ amplitude also influences the memristor’s weight modulation. Figure 5(c) shows the rectangular patches that represent the PPF and PTP properties in the relative conductance difference observed in the memristor with each pulse stimulation.The findings demonstrate that changes in conductance rise as the pulse height increases, resulting in rising PPF and PTP values.

This demonstrates that the amplitude of the pulse simulations as well as the pulse interval clearly influence the PPF and PTP behaviors. This further demonstrates how mimicking synaptic functions is a complicated process that depends on a number of variables. Furthermore, one of the essential synaptic functions in biological synapses is paired-pulse facilitation (PPF) [89]. The EPSC evoked by the spike is increased when the second spike closely follows a priori spike [90]. Our Pt/HfSiO_x_/TaN memristor emulates such PPF behavior. On the TE, two presynaptic spikes (–0.6 V, 10 µs) were delivered consecutively (pre-synapse). The typical excitatory postsynaptic currents (EPSCs), which have an inter-spike interval of 10 µs between them, are depicted in Fig. 5(d). The second presynaptic spike causes an increase in EPSC that is 1.27 times greater than the one caused by the first spike. This can be explained by how learning and memory decline over an extended period of time. Another possible explanation for this phenomenon is that the memristor can replicate the short-term plasticity (STP) observed in biological synapses, which involves changes in synaptic strength that occur over a short time scale (from milliseconds to seconds). Specifically, when the time interval between two electrical pulse stimuli is brief, the memristor may exhibit facilitation or depression, which is the enhancement or reduction of the synaptic response to the second stimulus, respectively. This effect can be seen as an increase or decrease in the response current of the artificial synapse [91]. As seen in Fig. 5(a), the PPF index gradually drops as the inter-time gap between the double pulses increases. The following is an expression for the PPF index [92]:

6 $$PPF\, Index= {A}_{2}/{A}_{1}\times 100 \%$$

where A_1_ and A_2_ indicate the first and second peaks, respectively. The A2/A1 value magnitude decreases as the pulse interval ($\varDelta t$) increases. As the pulse interval ($\varDelta t=0.1 ms$) lengthens, the amplitude of the A_2_/A_1_ value drops. The longest interval stimuli (Δt = 1.0 ms) have the lowest PPF ( 99%), whereas the shortest interval stimuli ($\varDelta t=0.1 ms$) have the highest PPF value ( 113%). The following details the mechanism. The first spike protons partially collect at the electrolyte interface when the second spike is administered shortly after the first spike. Because of the integrated effect, the accumulated protons will increase. As a result, the postsynaptic current will increase, and more induced electrons will be in the channel. While the summing effect vanishes and the A_2_/A_1_ value approaches 99% with very long $\varDelta t$, the protons generated by the first spike will diffuse back to the equilibrium location. Our memristors exhibit PPF behavior that closely resembles the PPF phenomenon observed in biological synapses. The device-to-device repeatability of Pt/a-HfSiO_x_/TaN memristor device was demonstrated to have excellent repeatability for PPF emulation with minimal device-to-device variations, as shown in Additional file 1: Fig. S6. The error bars display the device-to-device variations in the synaptic weight change. This confirms that the memristor device has short-term memory properties similar to biological synapses and has exceptional repeatability. The results could contribute to the development of an efficient neuromorphic computing system.

Various learning principles for long-term plasticity that mimic biological synapses must also be developed. This evaluation of the postsynaptic weight change in connection to the pre-synaptic pulse sequence is essential. The excitatory postsynaptic conductance (EPSC) of the Pt/a-HfSiO_x_/TaN memristor under varied voltage pre-spikes is used to gauge the analogies of a biological synapse further. This pre-synaptic pulse sequence alters the memristor conductance and has an impact on oxygen vacancy modulation. The EPSC response was measured following the application of an increasing number of stimulation pulses, from 1 to 50, with varying pulse amplitudes of − 0.6 V,–0.7 V, and − 0.8 V at the same pulse width (100 µs). Figure 5(g) represents the change in EPSC Gain versus the number of stimulation pulses. The EPSC Gain can be calculated by using the following equation [91]:

7 $$EPSC \,Gain= {A}_{n}/{A}_{1}$$

where ${A}_{n}$ and ${A}_{1}$ respectively refer to the amplitudes of EPSC after the last and 1st pulse from Fig. 5(f). The EPSC gain exhibited a clear potential rise trend and a saturated steady-state behavior at spike amplitudes of – 0.6 V, – 0.7 V, and – 0.8 V, respectively. The progressive increase in oxygen vacancy production within HfSiOx was thought to be the origin of these behaviors. Synaptic plasticity became saturated after 25 pulses due to the maximum number of oxygen vacancy generation. On the other hand, the progressively increasing concentration of oxygen vacancies production is responsible for the sequential increases in gain observed at – 0.6 V, – 0.7 V, and – 0.8 V concerning the number of spike pulses increases.

Similar to biological memory, this process predicts the strength of synaptic memory based on the number of repetitions [93]. The EPSC response of the memristor is also shown in Fig. 5(h), which increases the pulse count from 1 to 25 while adjusting the pulse widths of 100 µs and 200 µs at a constant pulse amplitude of -0.7 V, respectively. The relationship between EPSC and pulse count is seen in Fig. 5(i). Similar trends were seen, as seen in Fig. 5(g). After 10 pulses, the EPSC gain exhibits a modest saturation tendency. These findings demonstrated that an increase in EPSC with more pulses (at varying pulse amplitude and pulse width) clarifies the mimicking of bio-synapse response brought on by the relaxation process.

**Fig. 6:**
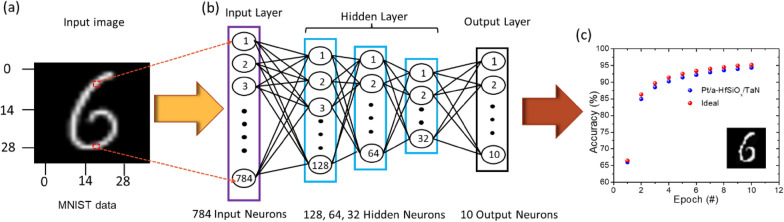
Simulation image classification using MNIST: **a** A handwritten “6” digit with 28 × 28-pixel input image used for the neural network training, **b** a schematic of the three-layer neural network with 784 input, 128, 64, 32 hidden, and 10 output neurons, respectively. **c** Recognition accuracy plotted against 100 training epochs. A classification accuracy of 94.6% is often attained. The inset graph displays the handwritten “6” digit recognition accuracy after using 10 learning epochs

We generated a synthetic neural network based on the devices as artificial synapses to carry out an image classification task to examine memristor computing capacity. As depicted in Fig. 6(a), we design a one input, three hidden and one output layered neural network in which every synaptic element adheres to the updating rule. Using the Modified National Institute of Standard and Technology (MNIST) handwritten digit dataset, a multilayer artificial neural network has been investigated to achieve supervised learning to assess the potential use of the Pt/a-HfSiO_x_/TaN memristor device in neuromorphic computing stimulation [94]. As seen in Fig. 6(b), the input images used were the handwritten “6” digit images from the MNIST collection, each measuring 28 × 28 pixels. As shown in Fig. 6(b), an artificial neural network of 784 input neurons, 128, 64, 28 hidden neurons, and 10 output neurons recognizes the handwritten “6” digit, and a digital output is obtained. A Pt/HfSiOx/TaN memristor was simulated in MNIST based on the experimental results of the 30 potentiation and 30 depression pulses (Fig. 4(i)). The accuracy of image categorization was assessed using 10, 0 0 0 test data after the network had been trained using 60, 0 0 0 training data. The growth of handwritten digit recognition accuracy over training epochs is depicted in Fig. 6(c). The memristor obtained a recognition accuracy of 94.6% for the 28 × 28 pixel image. These results imply that a-HfSiO_x_ memristors may have applications in artificial neuromorphic computing.

## Conclusion

In conclusion, Pt/a-HfSiO_x_/TaN memristor was fabricated to investigate the multilevel switching and in-memory computing systems through simulation of analog RS and neuromorphic synapses. The TaO_x_N_y_ interface layer is formed at the bottom interface between a-HfSiO_x_ and the high oxygen affinity TaN electrode, which is confirmed through TEM and XPS analyses. Improved RRAM performance, which has been empirically supported, includes lower set and reset voltage dispersion, a large memory window (10^3^), data retention property (10^4^ s), and stable repetitive switching behavior for 1000 cycles. Using the stopping reset voltage and current compliance limit, multilevel resistances were made possible. The conductance of a-HfSiOx based memristors can be controlled to exhibit long-term potentiation and long-term depression characteristics by adjusting the pulse height and pulse width, respectively. Additionally, the memristors have been shown to exhibit necessary synaptic functions for short-term plasticity, such as EPSC, PPF, PPD, PPF, and PTP. Additionally, neural network simulations achieve 94.6% of MNIST pattern recognition accuracy. These results support the excellent potential of a-HfSiO_x_ based memristor for use in large data memory storage and brain-inspired computing systems.

## Supplementary Information

**Additional file 1: Fig. S1**. displays a schematic illustration of the XPS and TEM analysis.** Fig. S2**. (a) XPS depth profile spectrum, (a) EDS, and (b) atomic % of the Pt/a-HfSiOx/TaN memristor device.** Fig.S3**. (a–e) Device-to-device (D1-D5) 100 consecutive I–V cycles of the Pt/a-HfSiOx/TaN memristor device.** Fig.S4**. (a–e) Device-to-device (D1-D5) endurance performance of the Pt/a-HfSiOx/TaN memristor device.** Fig. S5**. Device-to-device stability test: (a–c) 120 I-V characteristics, and (d–f) 200 cycles of endurance performance of the Pt/a-HfSiOx/TaN memristor device under different current compliance of 3 mA, 5mA and 10 mA, respectively.** Fig. S6**. PPF of the memristor as a function of the pulse interval with the pulse magnitude and width fixed at –1.0 V and 100 μs, respectively. The PPF measurement was conducted on 10 virgin devices for each pulse interval.

## Data Availability

The corresponding author can provide the datasets used and/or analyzed during the current study upon reasonable request.
