# Supplementary material for: Mimicking biological synapses with a-HfSiOx-based memristor: implications for artificial intelligence and memory applications
Source: Nano Converg. 2023 Jul 10;10:33. doi: 10.1186/s40580-023-00380-8 (PMC10333172; doi:10.1186/s40580-023-00380-8)
Supplement: Supplementary file 1 — Additional file 1: Fig. S1. displays a schematic illustration of the XPS and TEM analysis. Fig. S2. (a) XPS depth profile spectrum, (a) EDS, and (b) atomic % of the Pt/a-HfSiOx/TaN memristor device. Fig.S3. (a–e) Device-to-device (D1-D5) 100 consecutive I–V cycles of the Pt/a-HfSiOx/TaN memristor device. Fig.S4. (a–e) Device-to-device (D1-D5) endurance performance of the Pt/a-HfSiOx/TaN memristor device. Fig. S5. Device-to-device stability test: (a–c) 120 I-V characteristics, and (d–f) 200 cycles of endurance performance of the Pt/a-HfSiOx/TaN memristor device under different current compliance of 3 mA, 5mA and 10 mA, respectively. Fig. S6. PPF of the memristor as a function of the pulse interval with the pulse magnitude and width fixed at –1.0 V and 100 μs, respectively. The PPF measurement was conducted on 10 virgin devices for each pulse interval. [file 40580_2023_380_MOESM1_ESM.docx]

Mimicking biological synapses with a-HfSiOx-based memristors: Implications for artificial intelligence and memory applications

Muhammad Ismail^1^, Maria Rasheed^1^, Chandreswar Mahata^1^, Myounggon Kang^2^*, Sungjun Kim^1^*

^1^Division of Electronics and Electrical Engineering, Dongguk University, Seoul 04620, Republic of Korea

^2^ Department of Electronics Engineering, Korea National University of Transportation, Chungju-si 27469, Republic of Korea

* Corresponding author: [mgkang@ut.ac.kr](mailto:mgkang@ut.ac.kr) (M. Kang) and [sungjun@dongguk.edu](mailto:sungjun@dongguk.edu) (S. Kim)

Supplementary Information

Mimicking biological synapses with a-HfSiO_x_-based memristor: Implications for artificial intelligence and memory applications

Muhammad Ismail^1^, Maria Rasheed^1^, Chandreswar Mahata^1^, Myounggon Kang^2^*, Sungjun Kim^1^*

^1^Division of Electronics and Electrical Engineering, Dongguk University, Seoul 04620, Republic of Korea

^2^ Department of Electronics Engineering, Korea National University of Transportation, Chungju-si 27469, Republic of Korea

* Corresponding author: [mgkang@ut.ac.kr](mailto:mgkang@ut.ac.kr) (M. Kang) and [sungjun@dongguk.edu](mailto:sungjun@dongguk.edu) (S. Kim)


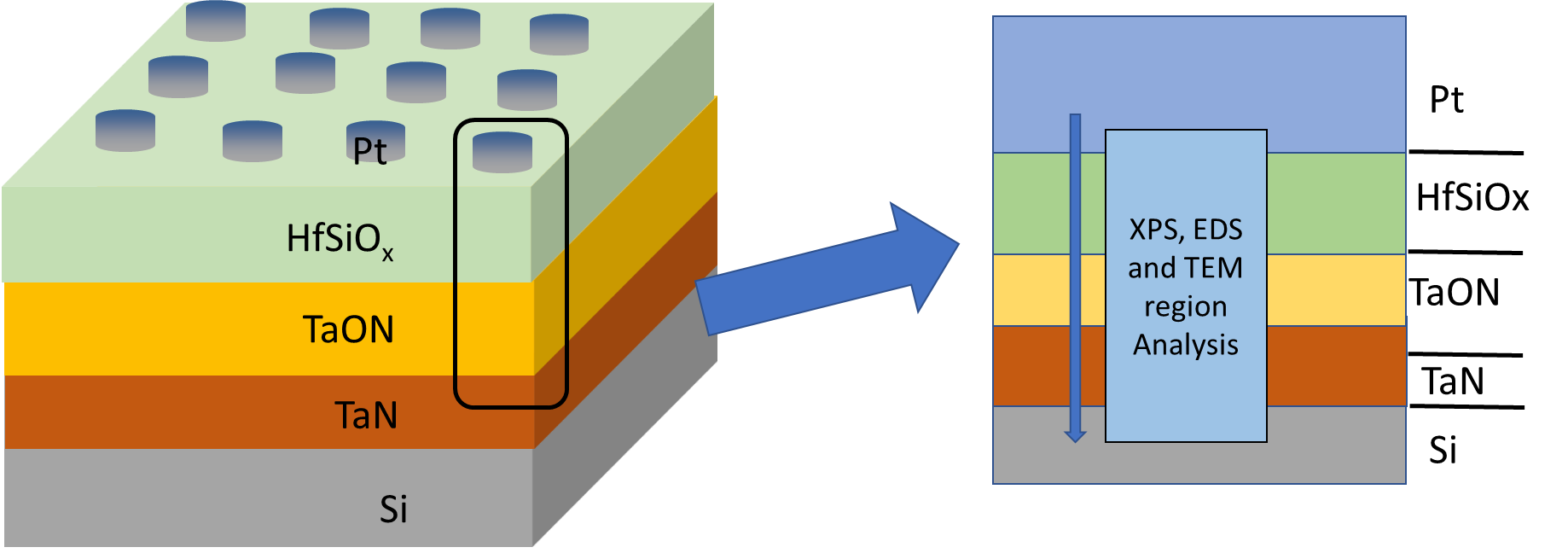


**Fig. S1**. displays a schematic illustration of the XPS and TEM analysis.


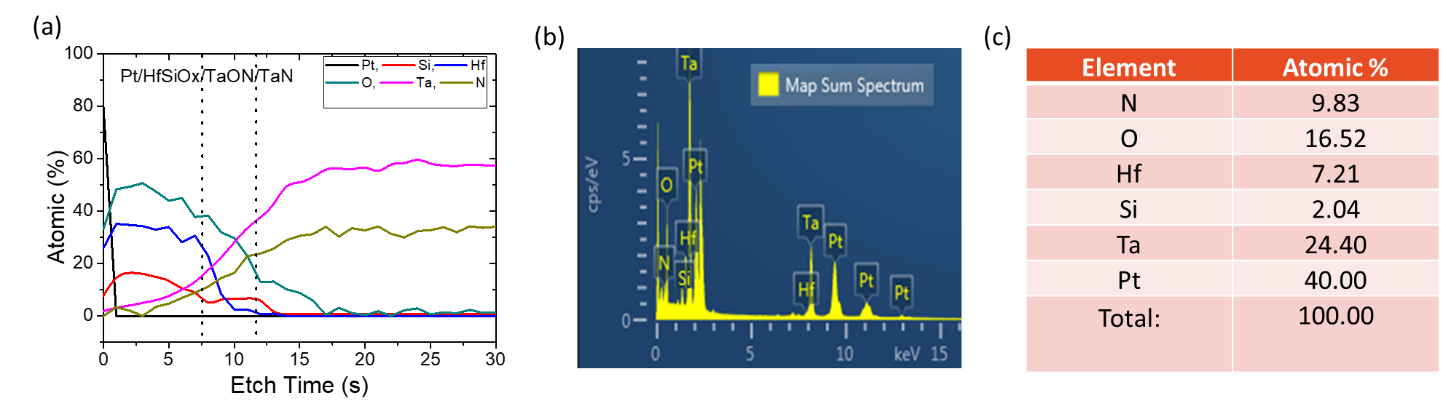


**Fig. S2.** (a) XPS depth profile spectrum, (a) EDS, and (b) atomic % of the Pt/a-HfSiO_x_/TaN memristor device.


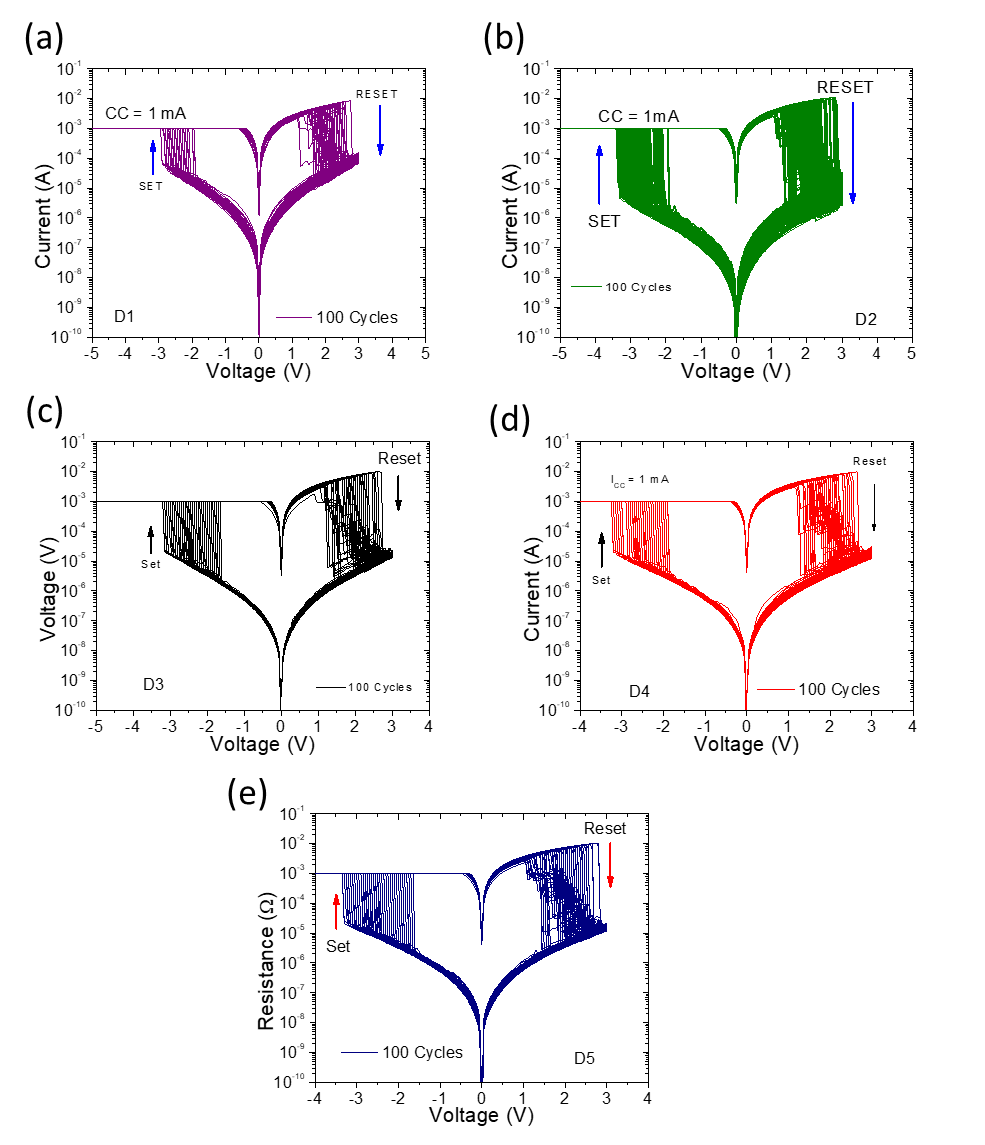


**Fig. S3.** (a-e) Device-to-device (D1-D5) 100 consecutive I–V cycles of the Pt/a-HfSiO_x_/TaN memristor device.


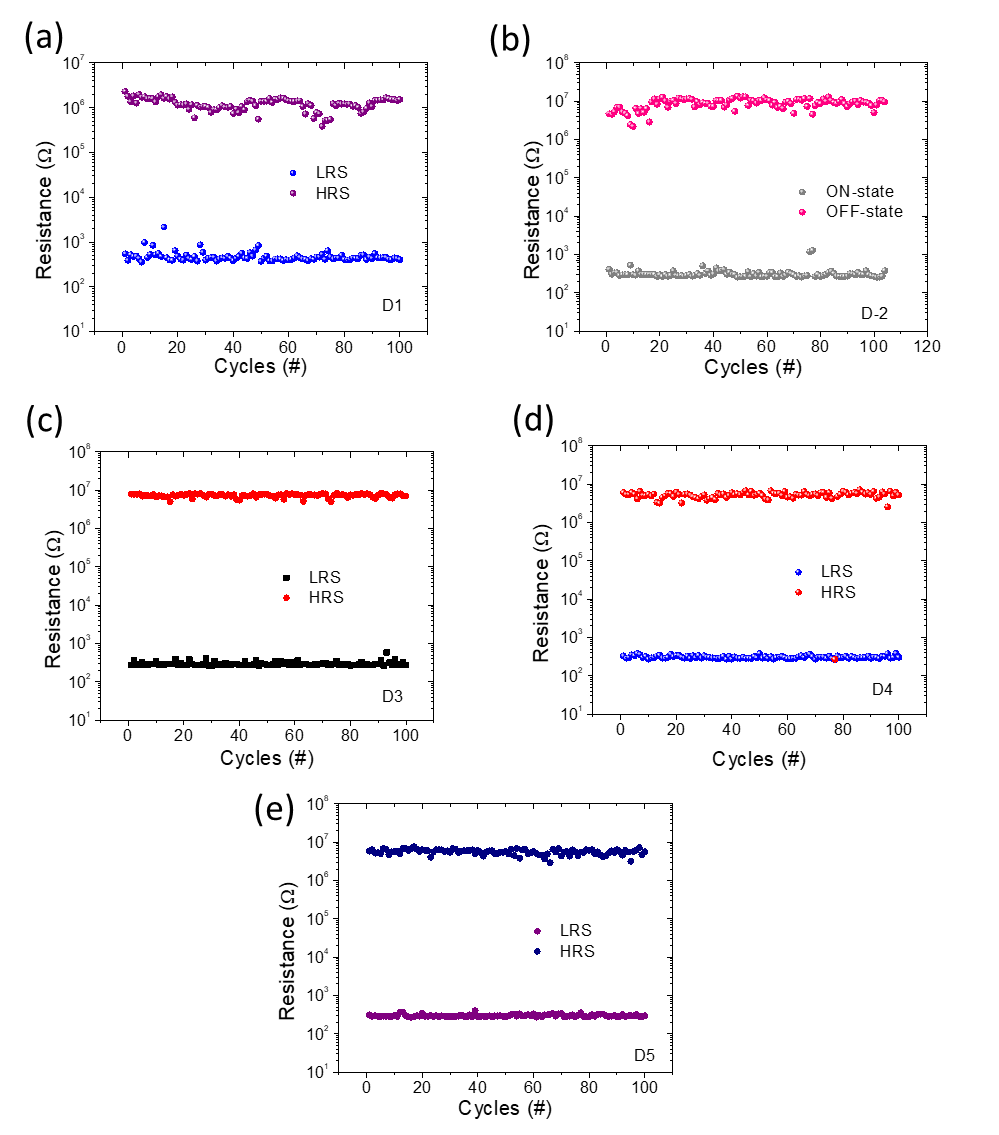


**Fig. S4.** (a–e) Device-to-device (D1-D5) endurance performance of the Pt/a-HfSiO_x_/TaN memristor device.


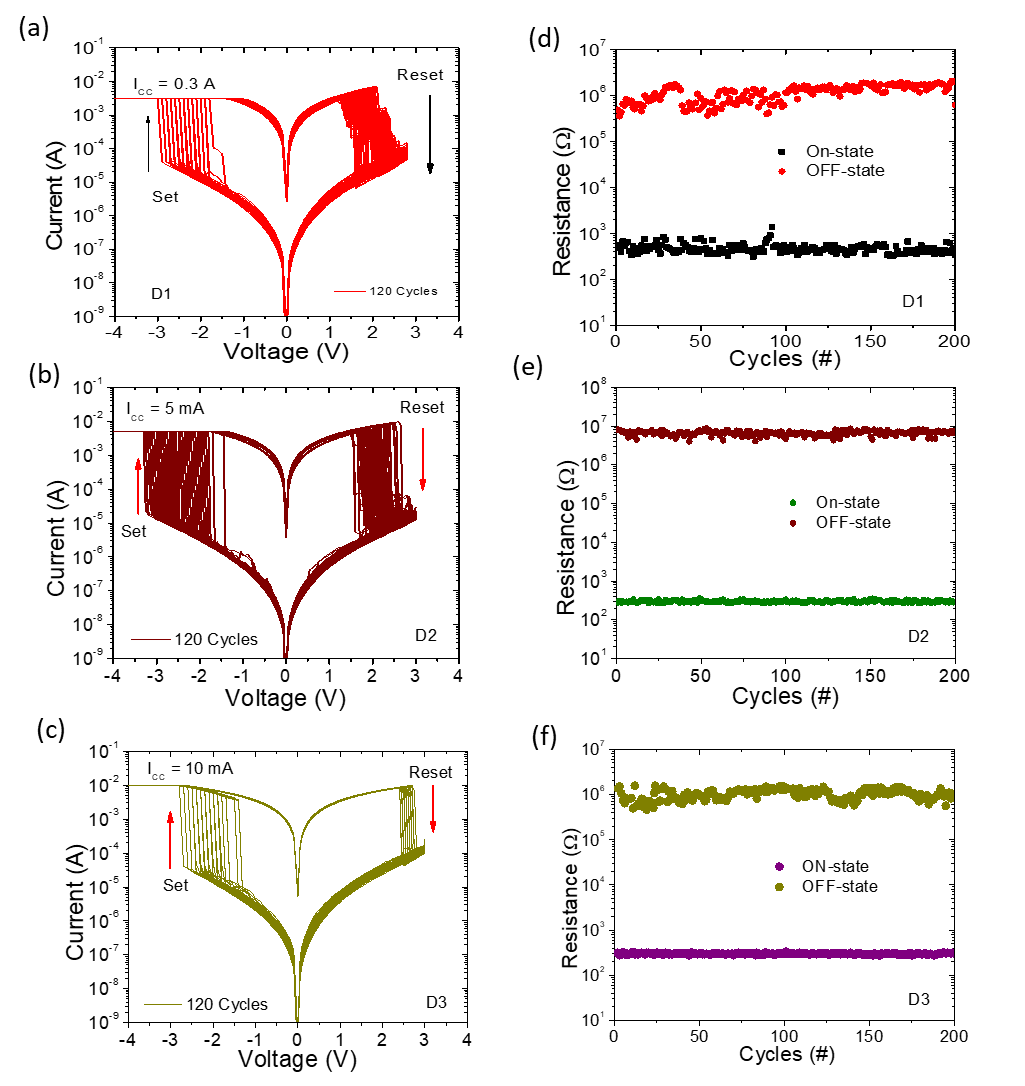


**Fig. S5.** Device-to-device stability test: (a-c) 120 I-V characteristics, and (d-f) 200 cycles of endurance performance of the Pt/a-HfSiO_x_/TaN memristor device under different current compliance of 3 mA, 5mA and 10 mA, respectively.

**Fig. S6.** PPF of the memristor as a function of the pulse interval with the pulse magnitude and width fixed at -1.0 V and 100 μs, respectively. The PPF measurement was conducted on 10 virgin devices for each pulse interval.
